# Introducing a novel catheter–tissue contact feedback feature in robotic navigated catheter ablation: Utility, feasibility, and safety

**DOI:** 10.1016/j.hroo.2020.04.003

**Published:** 2020-05-11

**Authors:** Anna Maria Elisabeth Noten, Tamas Géczy, Sing-Chien Yap, Zsuzsanna Kis, Tamas Szili-Torok

**Affiliations:** Department of Cardiology, Thoraxcenter, Erasmus MC, Rotterdam, The Netherlands

**Keywords:** Cardiac arrhythmia, Catheter ablation, Contact feedback, Remote magnetic navigation, Robotic navigation

## Abstract

**Background:**

The quality of catheter–tissue contact is one of the most important determinants of catheter ablation (CA) success. The absence of catheter–tissue contact feedback has been regarded a major limitation of remote magnetic navigation (RMN)–guided CA. The e-Contact module (ECM) is a novel feature designed for RMN that measures the quality of catheter–tissue contact.

**Objective:**

The purpose of this study was to describe the first clinical experience with this feature and to test its effect on procedural parameters and interference with other ablation equipment during CA procedures as well as its safety.

**Methods:**

This was a prospective, single-center, 2-phase study investigating ECM use during complex RMN procedures in 30 patients. Impact of ECM on procedural parameters was evaluated in the feasibility phase (FP), and its interference with other equipment was tested in the interference phase (IP) using pacing maneuvers at 3 randomly selected right atrial sites. Intracardiac electrograms were evaluated for disturbances by 2 independent electrophysiologists.

**Results:**

For FP, mean procedural time was 162 ± 66 minutes, fluoroscopy time 21 ± 9 minutes, and ablation time 34 ± 21 minutes. For IP, no significant differences in pacing capture or thresholds were found (ECM– vs ECM+: site 1: 2.05 vs 2.21 mA; *P* = .320; site 2: 2.15 vs 2.12 mA; *P* = .873; site 3: 2.51 vs 2.50 mA; *P* = .976). Electrogram disturbances did not significantly differ between ECM– and ECM+. No adverse events were reported.

**Conclusion:**

The ECM is a novel catheter–tissue contact technology designed for RMN-guided CA. Our study suggests that this feature is feasible and does not interfere with other electrophysiology equipment while maintaining an excellent safety profile.

**Key Findings**

▪ The e-Contact module (ECM) is a novel technology for assessing the quality of catheter–tissue contact in remote magnetic navigation (RMN)–guided catheter ablation.
▪ The quality of catheter–tissue contact is determined by 16 variables, including impedance measurements, cardiac-induced motion of the catheter, and magnetic torque data.
▪ This study demonstrated that the ECM is feasible, does not interfere with procedural parameters or other equipment used in the electrophysiology laboratory, and does not compromise the safety of RMN-guided catheter ablation procedures.

## Introduction

In radiofrequency (RF) catheter ablation (CA) procedures, catheters are placed in contact with myocardial tissue to diagnose and treat cardiac arrhythmias. An important determinant of successful ablation is appropriate contact between the tip of the ablation catheter and the targeted myocardial tissue.^1^^,^^2^ A variety of measures can be used to estimate the degree of contact, such as tactile feedback, catheter positioning on fluoroscopic and ultrasound imaging, catheter manipulation history, baseline impedance, impedance change, electrocardiogram amplitude assessment, and electrode temperature.^1^^,^^3^^,^^4^ In addition, complex electrical impedance and the associated electric coupling index have been used to assess contact^4^^,^^5^ and lesion size.^6^ However, all of these measures are still regarded as surrogate markers of effective lesion formation.^7^ More recently, contact force (CF) sensing catheters were introduced in manual-guided CA procedures. CF seemed to be a critical determinant of lesion size and transmurality.^10^, ^8^, ^9^ Initially, higher success rates^11^ and significantly less serious adverse events^12^ were reported using optimal CF in atrial fibrillation (AF) ablation studies compared to ablation with non-CF sensing catheters. However, more recent meta-analysis of randomized data showed that the availability of real-time CF feedback alone does not necessarily lead to improved clinical outcome or safety for CA of AF.^13^

One of the currently available RF CA techniques is remote magnetic navigation (RMN)–guided ablation. In RMN-guided RF ablation, the ablation catheter has a flexible distal tip and is navigated toward and held to the ablation surface only by magnetic force.^14^^,^^15^ In a myocardial phantom model, with simulated wall motion, RMN-guided ablation had deeper lesion dimensions compared to manual-guided ablation.^16^ In AF ablation, RMN was associated with faster modification of electrograms, suggesting transmurality compared to CA with optimized CF.^17^ In addition, RMN-guided ablation was reported to have a superior safety profile compared to manual ablation, with a very low incidence of cardiac perforation.^18^, ^19^, ^20^ However, a major limitation of RMN-guided ablation was the absence of a qualitative indicator of catheter–tissue contact. Recently, a novel contact technology—the e-Contact module (ECM)—was developed for RMN-guided CA. The ECM continuously monitors catheter–tissue contact using key ablation measures to gauge the quality of contact between the magnetically guided catheter and myocardial tissue. The present study aimed to describe this novel feature and to investigate its feasibility, its effect on procedure parameters, its interference with other electrophysiology (EP) equipment, and its safety.

## Methods

### Study design

This study was designed as a single-center, prospective, observational, 2-phase study. The 2 study phases were a feasibility test phase (FP) and an interference test phase (IP). In the FP, complex RMN-guided CA procedures were performed with ECM guidance. The ablation procedures were performed per standard of care. Procedural parameters were recorded and compared with literature.

The IP consisted of a pacing protocol conducted either at the beginning or at the end of the procedure, based on the operator’s preference. The IP started with composition of an electroanatomic map of a portion of the right atrium while patients were in sinus rhythm. Three sites in the right atrium were randomly selected and tagged to test pacing parameters. Regions with previous or present ablations were avoided to ensure pacing capture. If cavotricuspid isthmus (CTI) ablation was performed before the IP study protocol was conducted, a 3-cm margin was reserved between the pacing sites and the ablated area. After disconnection of ECM (ECM–), nonaggressive pacing (cycle length 500–700 ms; pulse width 2.0 ms) was conducted at the 3 marked sites to determine pacing capture and pacing thresholds. After ECM reconnection (ECM+), the 3 sites were relocated and the pacing protocol was conducted again. All intracardiac electrograms were recorded. Postprocedure, the operating electrophysiologist completed a data questionnaire evaluating the feasibility of the ECM. Outside of the EP laboratory, 2 independent, blinded, highly experienced electrophysiologists evaluated the intracardiac recorded electrograms for differences and disturbances caused by ECM connection and scored the severity as mild (not affecting electrogram evaluation); moderate (some interference with electrogram evaluation); or severe (evaluation of electrogram impossible).

### Study population

The study recruited 30 consecutive patients who underwent a CA procedure by RMN using ECM for treatment of cardiac arrhythmia beginning April 1, 2017. All types of cardiac arrhythmias were valid for inclusion in the study, and all patients were eligible for CA based on current guidelines and insights.^21^^,^^22^ All CA procedures were performed in accordance with institutionally approved local medical treatment protocols. The study was approved by the institutional review boards for human research (ClinicalTrials.gov Identifier: NCT03103945).

### Exclusion criteria

Patients who were not in sinus rhythm at either the beginning or the end of the procedure were considered not eligible for inclusion. Patients had to be ≥18 years to participate in the study. Presence of cardiac thrombus was a contraindication for CA as well as study participation.

### Procedural protocol

All procedures were performed using the Niobe ES RMN system (Stereotaxis, Inc, St. Louis, MO), the CARTO 3-dimensional electroanatomic mapping system (Biosense Webster Inc, Diamond Bar, CA), and the Navi-Star RMT ThermoCool ablation catheter (Biosense Webster). The Ablation History feature in the Navigant software (Stereotaxis) was used during all procedures. Ablation History provides a visual display of the history of the catheter's power output and duration of energy application at each location in the map during ablation. Based on the operator’s preference, it was allowed to use continuous dragging of the catheter while ablating rather than applying point-by-point lesions. Ablation was performed using the following RF settings: left atrial anterior wall: 50 W, flow 17 mL/min, maximum 43°C; left atrial posterior wall: 45 W, flow 17 mL/min, maximum 43°C; right ventricular outflow tract: 45–50 W, flow 20 mL/min, maximum 43°C; aortic cusp: 20 W gradually increasing to 45–50 W, flow 30 mL/min, maximum 43°C; right ventricle: 40–45 W, 20 mL/min, maximum 43°C; left ventricle: 50–55 W, 30 mL/min, maximum 43°C.

### Data collection

Baseline demographic and clinical characteristics were collected from the institutional electronical medical files (HiX version 6.1). Procedural and pacing data were collected during the procedure using data collection sheets. Intracardiac electrograms were recorded by the EP-WorkMate (St. Jude Medical Inc, St. Paul, MN) and Odyssey Cinema (Stereotaxis) systems. The procedural data collection forms contained a questionnaire for the operating electrophysiologist, from which information on acute adverse events was collected as well.

### ECM: Description of technology

**Figure 1:**
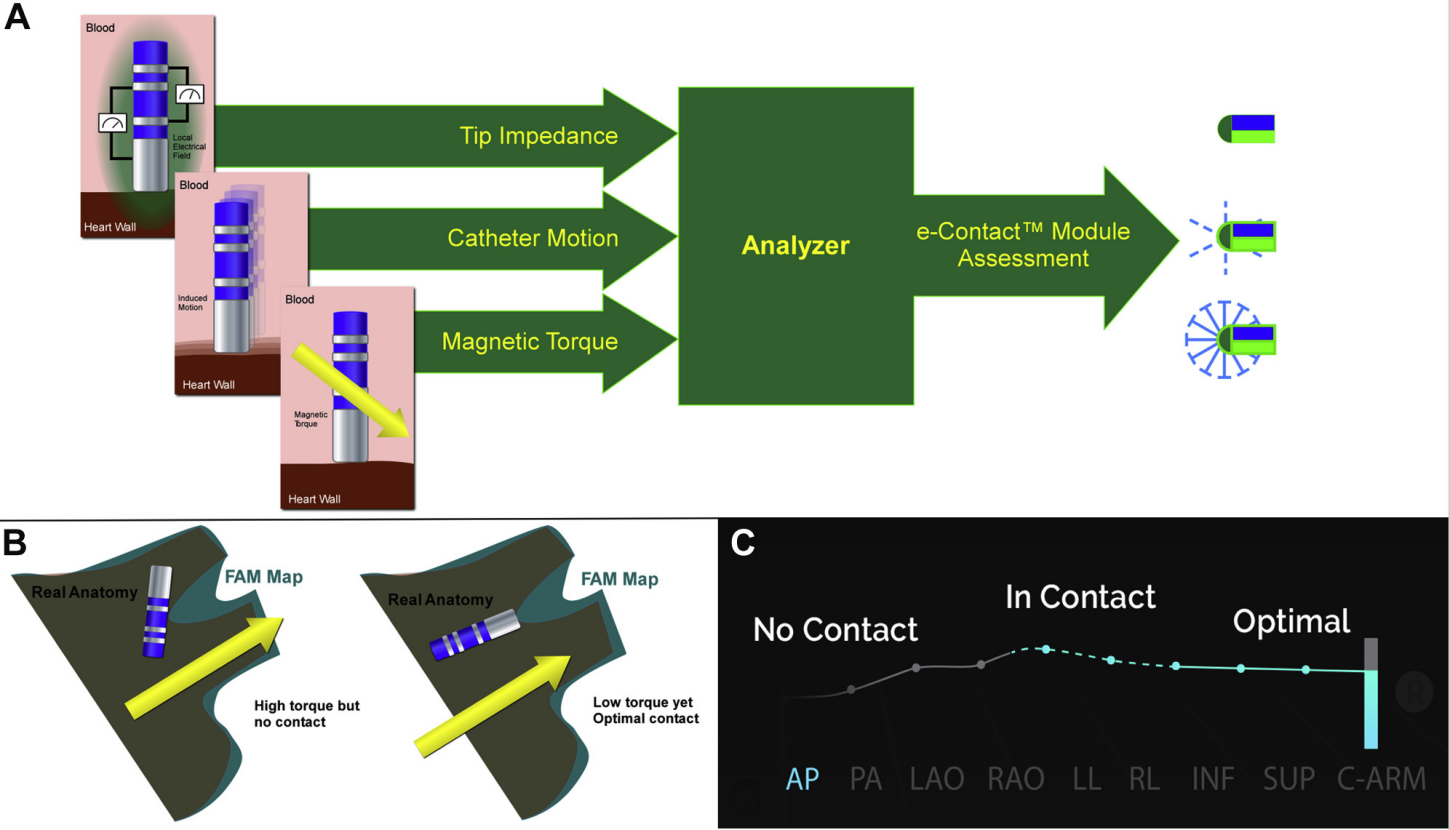
The e-Contact module (ECM). **A:** Schematic overview of the 3 types of data incorporated in the ECM. The ECM analyzes impedance measurements, externally (cardiac) induced motion of the catheter, and magnetic torque data. The result of contact assessment is displayed to the user as a starburst near the catheter tip. For any contact, the starburst is small. When the contact is more optimal, the starburst is bolder. **B:** Magnetic torque data alone are insufficient to establish whether the catheter tip is in good contact with cardiac tissue. High torque could be caused by contact of the catheter shaft with cardiac tissue instead of the catheter tip. On the contrary, the catheter tip could be in optimal contact with myocardial tissue as well as being perfectly aligned with the magnetic field, resulting in low torque. **C:** Contact assessment is also displayed to the user on the contact tracing. For suboptimal contact, the contact tracing shows as a *dotted line.* For optimal contact, the contact tracing becomes a *solid line.* AP = anteroposterior; FAM = fast anatomic mapping; INF = inferior; LAO = left anterior oblique; LL = left lateral; PA = posteroanterior; RAO = right anterior oblique; RL = right lateral; SUP = superior.

The Niobe ES RMN system (Stereotaxis) is a medical platform designed for EP and interventional procedures. The RMN system uses 2 permanent magnets, one on each side of the patient, to remotely guide the movement of the distal tip of compatible ablation catheters via magnetic fields. This technique has been described and validated extensively elsewhere.^14^^,^^15^^,^^23^ ECM is a recently developed hardware and software module that is compatible with the Niobe ES RMN system. The ECM box is a hardware unit that is an interface between the cardiac catheter and the CARTO Patient Interface Unit (Biosense Webster). A schematic image explaining the connection of the ECM box to the Niobe ES system and ablation catheter is shown in Supplemental Figure 1. The ECM hardware facilitates impedance measurement between the electrodes at the catheter tip. The ECM software algorithm incorporates 16 types of data from 3 categories to determine whether the catheter is in contact with cardiac tissue by analyzing electrical impedance measurements, cardiac-induced motion of the catheter tip, and the torque being applied by the magnetic field. These components are shown schematically in Figure 1.

In order to determine catheter–tissue contact, the ECM analyzes both unipolar and bipolar impedance measurements. Optimal contact means that conditions are present for good energy transfer between the tip and myocardial tissue. The better the quality of the connection between the tissue and the catheter tip, the more efficiently RF energy flows into the tissue during ablation. A higher catheter-to-tissue impedance ratio corresponds with a higher ratio of energy absorbed by the tissue to energy lost to the blood. To confirm the different threshold levels of contact, qualitative assessments based on observations during preclinical studies were made while visually observing contact using intracardiac ultrasound. More stable contact was observed in association with delta bipolar impedance changes >5 Ω above the impedance of the blood pool. The terms “in contact” and “optimal” were chosen to represent this observed difference between just touching and stable contact. No specific outcomes or lesion delivery characteristics are claimed for the levels of contact. In addition to the impedance change information, cardiac-induced motion of the catheter is incorporated in the contact assessment. Compared to manually guided catheters, the RMN-guided catheter has a unique characteristic: when the tip is not in contact with the cardiac wall, it settles into a minimum energy configuration that aligns with the magnetic field. If the catheter is moved out of this minimum energy position, it induces a higher energy content dynamic state. Therefore, the catheter must be reacting to that external force. By measuring the induced motion caused by external forces acting on the catheter, the dynamic of the energy of the catheter can be determined. Magnetic torque results when there is a difference in the alignment of the catheter with the magnetic field. These magnetic torque data are analyzed by the ECM. When the catheter comes in contact with cardiac tissue, the cardiac wall moves the catheter out of alignment with the magnetic field, resulting in magnetic torque. Therefore, a high torque state is an indication of contact as well. The externally induced motion and torque data alone are insufficient to establish whether the catheter tip is in good contact with cardiac tissue, as they could be influenced by contact of the catheter shaft with cardiac tissue instead of the catheter tip (Figure 1B). Thus, impedance measurements are critically important when determining adequate catheter–tissue contact.

**Figure 2:**
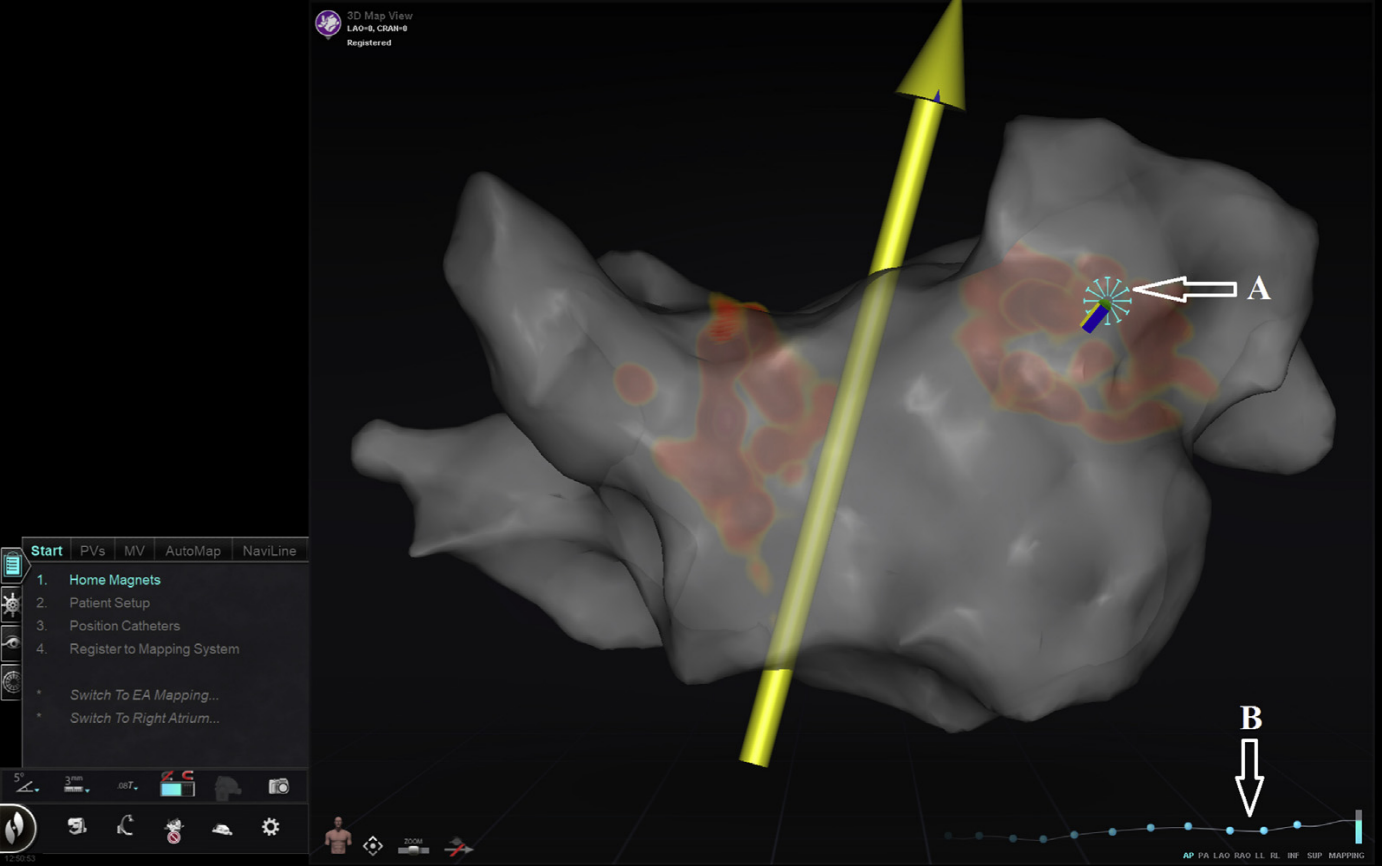
Contact feedback by the e-Contact module (ECM). Screenshot of the CARTO screen on which an anatomic map of the left atrium (LA) during a catheter ablation procedure for atrial fibrillation is shown, with the ablation catheter in place. Contact feedback by the ECM is displayed to the operator on this screen, indicating that the catheter is in “optimal” contact with LA tissue by displaying a dense starburst at the catheter tip (*A*) and by the dense line on the contact tracing (*B*).

The ECM system uses a visual tristate indicator for catheter–tissue contact. If the contact indicator is not visualized, the tip of the catheter is in the blood. As the catheter comes in contact with the tissue, a starburst indicator displays near the catheter tip, and a blue line appears on the contact indication tracing. For minimal contact, the starburst is small, and the contact tracing is displayed as a blue dotted line. For optimal contact, the starburst is bolder, and the contact tracing becomes a solid line (Figure 1). The ECM output is incorporated into and displayed to the operator on the CARTO screen (Figure 2). Of note, in contrast to CF sensing catheters, the ECM does not provide the amount of CF applied. Instead, the ECM determines the quality of contact with cardiac tissue based on the parameters described.

### Statistical analysis

For normally distributed continuous variables, mean ± SD were calculated. Median (interquartile range [IQR]) was computed for continuous variables with a non-normal distribution. Descriptive statistics for categorical data are expressed as absolute number (percentage). Comparison between groups of normally distributed continuous variables was made by the paired-samples Student *t* test. For non-normal distribution of data, the Wilcoxon signed-rank test was used. The χ2 test was used for comparison of categorical data. For electrogram evaluations, interobserver agreement was assessed using Cohen kappa analysis. Excellent, moderate, fair, and poor agreement were defined as κ-coefficients of 1.00–0.61, 0.60–0.41, 0.40–0.21, and <0.20, respectively.^24^ Two-sided *P* <.05 (2-tailed) was considered significant. Data were analyzed using SPSS 24.0 (SPSS Inc, Chicago, IL).

## Results

A total of 30 patients were included in the study. Descriptive demographic and clinical data are summarized in Table 1. Median age was 59.5 years (IQR 48.8–65.3), and 40% of participants were female. Overall, patients had reasonable cardiac function (mean ejection fraction 53.2% ± 7.4). EP medical history of the patients is given in Table 2. Most of the patients had a history of AF (n = 26 [87%]). Some patients had several types of cardiac arrhythmias in their medical history.

**Table 1:** Baseline patient demographic and clinical data (N = 30)

	
Age (y)	59.5 (48.8–65.3)
Female	12 (40)
BMI (kg/m^2^)	28.0 ± 4.7
Hypertension	11 (37)
Dyslipidemia	4 (13)
Diabetes mellitus	2 (7)
Ischemic heart disease	2 (7)
Dilated cardiomyopathy	2 (7)
OSAS	1 (3)
CHA_2_DS_2_-VASc score	
0	11 (37)
1	11 (37)
≥2	8 (27)
Beta-blocker	14 (47)
Amiodarone	8 (27)
Flecainide	3 (10)
Sotalol	8 (27)
Calcium antagonist	3 (10)
Anticoagulation	
None	4 (13)
Coumadin	9 (30)
DOAC	17 (57)
LVEF (%)	53.2 ± 7.4
LVEF ≥55%	18 (60)
LVEF 45%–54%	9 (30)
LVEF 30%–44%	3 (10)
LVEF <30%	0 (0)
LA volume (mL)	85.8 ± 23.9
LA size (mm)	41.8 ± 6.4

Values are given as median (interquartile range), n (%), or mean ± SD.

BMI = body mass index; DOAC = direct-acting oral anticoagulation; LA = left atrium; LVEF = left ventricular ejection fraction; OSAS = obstructive sleep apnea syndrome.

**Table 2:** Baseline EP medical history (N = 30)

	
Previous EP procedure	9 (30)
Previous PVI	8 (27)
Atrial fibrillation	26 (87)
Paroxysmal	18 (60)
Persistent	8 (27)
Atrial fibrillation duration (y)	4.11 (1.33–7.08)
Atrial flutter	6 (20)
Atrial flutter duration (y)	3.00 (0.60–10.75)
Other SVT	1 (3)
Other SVT duration (y)	28.00 (28.00–28.00)
PVC	2 (7)
PVC duration (y)	2.33 (0.92–3.75)

Values are given as n (%) or median (interquartile range).

Note: Some patients experienced various arrhythmias.

EP = electrophysiology; PVC = premature ventricular contraction; PVI = pulmonary vein isolation; SVT = supraventricular tachycardia.

### FP results

Pulmonary vein isolation (PVI) was the most frequently performed procedure (n = 21 [70%]) (Table 3). Other ablation procedures included in the study were PVI combined with manual CTI ablation (n = 3 [10%]); atypical atrial flutter (aAFL) or atrial tachycardia (AT) ablation (n = 3 [10%]); and premature ventricular complex (PVC) ablation (n = 3 [10%]). Mean procedural time of all procedures was 162 ± 66 minutes, total fluoroscopy time 21 ± 9 minutes, and total ablation time 34 ± 21 minutes. PVI was successful in all patients (n = 24 [100%]), as demonstrated by either entry or exit block of spontaneous or paced beats. CTI was successful in all patients (n = 3 [100%]), as demonstrated by bidirectional block. Termination of the tachycardia was observed in 15 of 22 patients (68%) with sustained AF or AT during ablation. All PVC ablations in the 3 patients were performed in the right ventricle: 1 in the right ventricular outflow tract, 1 at the lateral border of the tricuspid annulus, and 1 at the septal insertion of the moderator band. PVCs terminated during the procedure in 2 patients (67%).

**Table 3:** Procedural data

	PVI (n = 21 [70%])	PVI + CTI (n = 3 [10%])	aAFL/AT (n = 3 [10%])	PVC (n = 3 [10%])	All (N = 30 [100%])
Procedural time (min)	171 (135–215)	185 (112–185)	80 (72–150)	163 (80–163)	162 ± 66
Fluoroscopy time (min)	22 (19–28)	27 (18–27)	30 (16–44)	4 (0–4)	21 ± 9
Ablation time (min)	34 (21–51)	47 (12–47)	32 (14–32)	10 (9–10)	34 ± 21
No. of applications	17 (11–24)	27 (16–27)	13 (4–21)	12 (7–12)	17 (11–34)
Sustained tachycardia during ablation	16 (76)	3 (100)	3 (100)	NA	22 (73)
Termination of tachycardia	10 (63)	3 (100)	2 (67)	2 (67)	17 (68)

Values are given as median (interquartile range), mean ± SD, or n (%).

aAFL = atypical atrial flutter; AT = atrial tachycardia; CTI = cavotricuspid isthmus; NA = not applicable; PVC = premature ventricular contraction; PVI = pulmonary vein isolation.

### IP results

**Figure 3:**
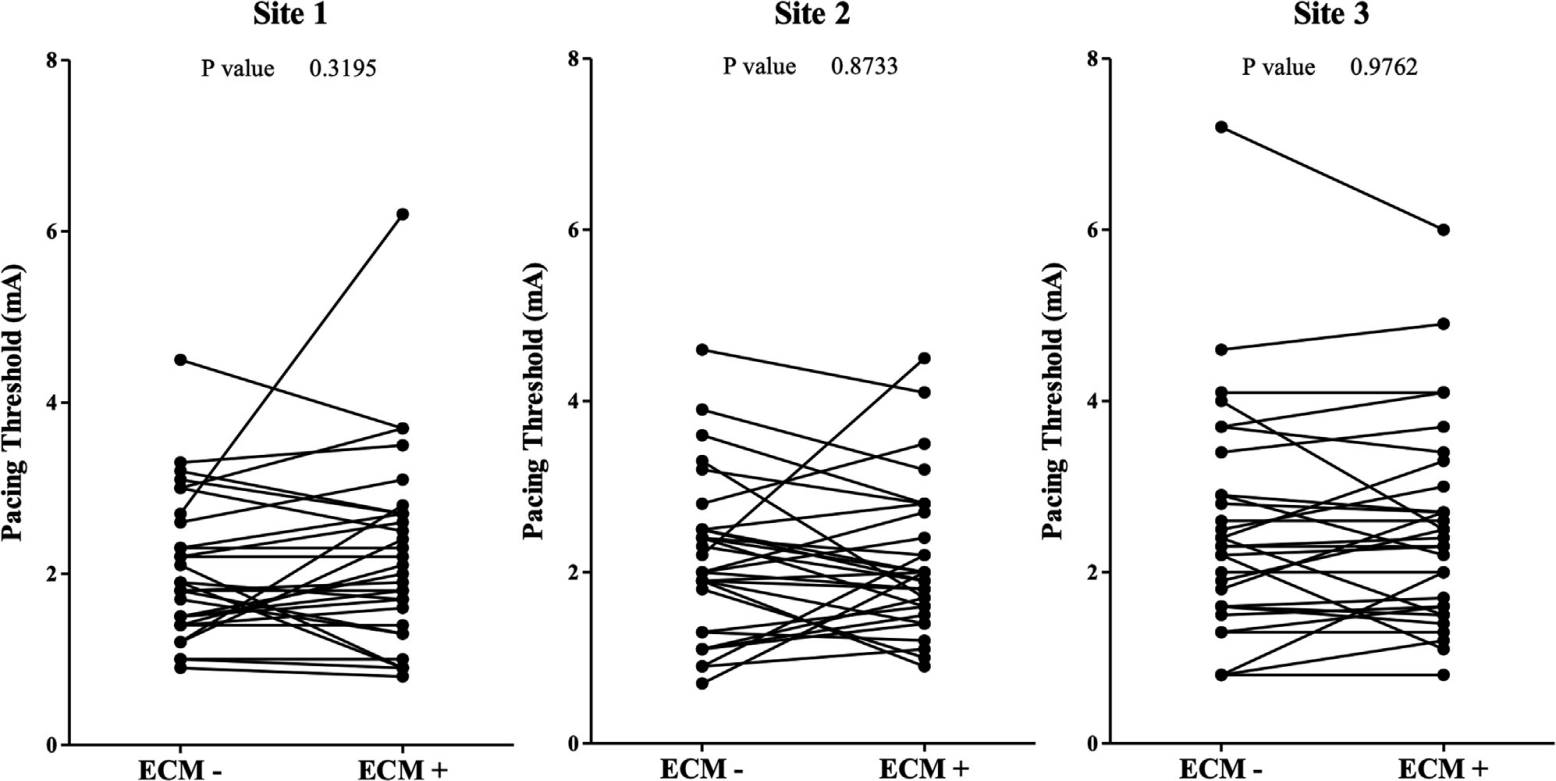
Comparison of pacing thresholds recorded at 3 randomly selected and tagged sites of the right atrium. Pacing thresholds were compared using the paired-samples Student *t* test. No significant differences caused by e-Contact module (ECM) connection were observed.

Results of the pacing protocol are given in Table 4 and Figure 3. At all sites, pacing resulted in capture regardless of ECM connection (100% vs 100%; *P* = 1). No significant differences in mean pacing threshold were found between ECM– and ECM+ at all pacing sites (site 1: 2.05 vs 2.21 mA; *P* = .320; site 2: 2.15 vs 2.12 mA; *P* = .873; site 3: 2.51 vs 2.50 mA; *P* = .976). Results of the evaluation of intracardiac recorded electrograms (N = 87) are listed in Table 5. In 1 case, the electrogram recordings were lost (n = 3). The first electrophysiologist identified disturbances on 1 ECM– electrogram (1%) and 3 ECM+ electrograms (3%) (*P* = .312). However, the second electrophysiologist identified disturbances on 4 ECM– electrograms (5%) and 3 ECM+ electrograms (3%) (*P* = .700). According to both electrophysiologists, the scored severity of disturbances was not significantly different between ECM– and ECM+. Cohen kappa analysis was run to determine whether there was agreement in electrogram disturbance and disturbance severity scores between the 2 electrophysiologists. Both variables yielded excellent interobserver agreement (disturbance: κ = 0.719; *P* <.001; disturbance severity score: κ = 0.628; *P* <.001) (Supplemental Tables 1 and 2).

**Table 4:** Pacing capture and thresholds (N = 180)

	ECM not connected (N = 90)	ECM connected (N = 90)	*P* value
Site 1			
Pacing capture	30 (100)	30 (100)	1
Pacing threshold (mA)	2.05 ± 0.83	2.21 ± 1.12	.320
Site 2			
Pacing capture	30 (100)	30 (100)	1
Pacing threshold (mA)	2.15 ± 0.93	2.12 ± 0.87	.873
Site 3			
Pacing capture	30 (100)	30 (100)	1
Pacing threshold (mA)	2.51 ± 1.34	2.50 ± 1.18	.976

Values are given as n (%) or mean ± SD unless otherwise indicated.

ECM = e-Contact Module.

**Table 5:** Intracardiac electrograms (N = 174)

	ECM not connected (N = 87)	ECM connected (N = 87)	*P* value
Disturbance noted			
Electrophysiologist 1	1 (1)	3 (3)	.312
Electrophysiologist 2	4 (5)	3 (3)	.700
Disturbance severity∗ (mild/moderate/severe)			
Electrophysiologist 1	1 (1)/0 (0)/0 (0)	2 (2)/1 (1)/0 (0)	.507
Electrophysiologist 2	3 (4)/1 (1)/0 (0)	2 (2)/0 (0)/1 (1)	.531

Values are given as n (%) unless otherwise indicated.

ECM = e-Contact module.

∗ Disturbance was divided into 3 categories: mild (not affecting electrogram evaluation); moderate (some interference with electrogram evaluation); and severe (evaluation of electrogram impossible).

### Results of questionnaire

According to the operating electrophysiologists, the ECM gave accurate real-time information of contact of the ablation catheter with myocardial tissue. Optimal contact (visualized as a dense starburst) was observed when the catheter was placed actively in contact with the atrial wall, whereas no starburst was seen when the catheter was placed in the blood pool. No technical problems caused by ECM connection were reported. In our study, neither minor nor serious acute adverse events were reported.

## Discussion

This is the first study to describe the ECM incorporated into the Stereotaxis RMN system. Our data suggest that the ECM can be safely and effectively implemented in RMN-guided CA procedures. The ECM is feasible and may contribute to procedural efficiency. Our study also demonstrates that the ECM does not interfere with procedural parameters or other equipment used in the EP laboratory, and it does not compromise the safety of RMN-guided CA procedures.

Numerous studies have been conducted in search of the best indicator of catheter–tissue contact.^1^, ^2^, ^3^, ^4^, ^5^, ^6^, ^7^ Impedance measurements have been proposed, but these measurements alone have always been regarded as surrogate measures.^7^ The contact quest came to its highpoint with the development of CF sensing catheters for manual-guided RF CA procedures. In manual AF ablation, optimal CF initially was reported to have high acute success rates,^11^ favorable long-term outcomes, and an improved safety profile compared to non-CF sensing catheters.^12^^,^^25^ Indeed, insufficient CF has been linked to an increased risk of electrical reconnection and arrhythmia recurrence, whereas excessively high CF has been associated with cardiac perforation.^26^ However, more recent meta-analysis of randomized data showed that the availability of real-time CF feedback alone does not necessarily lead to improved clinical outcome or safety, thus introducing controversy about the benefits of this technology.^13^^,^^27^^,^^28^ The introduction of CF sensing catheters to ventricular tachycardia ablation did not provide additional benefit over non-CF catheters,^29^ and most studies reported superiority of RMN-guided over manual (CF/non-CF)–guided ablation.^29^, ^30^, ^31^ As described, RMN uses magnetic force to guide catheters to the targeted myocardial tissue. CF sensing technology is not compatible with RMN, as it would unnecessarily increase the stiffness of the magnetically guided catheter. However, in an experimental model, magnetic fields of 0.08 and 0.10 T had stable catheter CFs, with an average of 6*g* measured without a sheath, which increased to 20*g* with use of a long sheath advanced to the entrance of the chamber of interest.^32^ The highly flexible shaft of the magnetic-guided catheter allows for multiangle and sharp contortions.^32^ The flexibility of the RMN-guided catheter is believed to be the origin of the improved safety profile of RMN-guided ablation and the low numbers of associated cardiac perforation.^18^ However, the lack of qualitative contact assessments for RMN-guided CA procedures was regarded a major limitation of the technique. The novel contact assessment technology described in this study was specifically developed for RMN. In contrast to parameters used in the past, it estimates contact using 16 variables from 3 categories, providing more accuracy. The contact assessment by the ECM was shown not to negatively influence pacing or disturb intracardiac electrograms. The current study demonstrated that the ECM does not interfere with other EP equipment and is safe to use. In addition, the ECM hardware and software update are easy to implement. Therefore, we suggest implementing the ECM in RMN-guided ablation as standard of care.

### Effects on efficiency

In our study, the ECM provided accurate real-time information on contact of the ablation catheter tip with myocardial tissue, leading to efficient RMN-guided CA procedures. Bauernfeind et al^18^ compared procedural parameters between RMN-guided and manual-guided CA ablation procedures. They reported mean procedural time of 248 ± 59 minutes and fluoroscopy time of 44 ± 17 minutes in RMN-guided AF ablation. For aAFL/AT ablation, average procedural time was 188 ± 51 minutes and fluoroscopy time was 37 ± 23 minutes.^18^ Two meta-analyses on RMN-guided AF ablation^19^^,^^20^ and a study by Kim et al^33^ reported similar results with long procedural times. The present study demonstrated shorter AF/aAFL/AT procedural and fluoroscopy times compared to these earlier studies. In our opinion, these differences can be attributed to technological advances made in RMN technology by implementation of the ECM. Integration of the IP study protocol into the procedures has extended the reported procedural times. Therefore, no comparison with historical data was performed. Future research is needed to investigate the impact of the ECM on procedural efficiency.

### Effects on outcome

Theoretically, the most important clinical advantage of this novel modality is improved ablation outcome. Because of contact feedback, the operator can optimize ablation catheter positioning before starting energy application, thus preventing inadequate lesion formation, which could become an arrhythmogenic substrate in the future. Also, contact feedback prevents gap persistence in the ablation lines, as the operator can monitor wall contact while ablating. Lesion assessment remains a determination made by the physician based on experience and data presented by various systems and not by ECM alone. The specific quantitative implications of the amount of impedance change on lesion formation is a matter on ongoing research. Comparative studies are warranted to investigate the impact of technological advancements, such as the ECM, on lesion formation and outcome based on the levels of contact displayed by the ECM.

### Study limitations

One limitation of this study is the relatively small study sample, which predominantly consisted of AF ablations. However, because the data assessed by the ECM technology are obtained equally from all cardiac regions, different findings are not expected during non-AF arrhythmia ablation. We used subjective measurements to evaluate electrogram disturbance. However, the electrograms were evaluated by 2 independent electrophysiologists, which yielded excellent interobserver agreement. Regarding our study objectives, the most important finding is that almost no disturbances were found by either of the electrophysiologists. Therefore, we believe that observer bias is negligible.

## Conclusion

The ECM—a novel contact technology designed for RMN-guided CA—is safe, feasible, and does not interfere with surrounding technologies in the EP laboratory. For the first time, the quality of catheter–tissue contact can be determined for RMN-guided CA of cardiac arrhythmias.
