# Supplementary material for: Introducing a novel catheter–tissue contact feedback feature in robotic navigated catheter ablation: Utility, feasibility, and safety
Source: Heart Rhythm O2. 2020 May 11;1(2):103–10. doi: 10.1016/j.hroo.2020.04.003 (PMC8183953; doi:10.1016/j.hroo.2020.04.003)
Supplement: Supplemental Material [file mmc1.docx]

**Supplemental material**

**Introducing a novel catheter-tissue contact feedback feature in Robotic Navigated Catheter Ablation: Utility, Feasibility and Safety**

Anna Maria Elisabeth Noten MD^1^, Tamas Géczy MD PhD^1^, Sing-Chien Yap MD, PhD^1^, Zsuzsanna Kis MD^1^, Tamas Szili-Torok MD, PhD^1^, FHRS

1: Department of Cardiology, Thoraxcenter, Erasmus MC, Rotterdam, the Netherlands

**Short title:** Contact Feedback for Robotic Ablation

Corresponding author:

Tamas Szili-Torok, MD, PhD

Thoraxcenter, Department of Cardiology, Erasmus MC

Postbus 2040, 3000 CA Rotterdam, The Netherlands

E-mail to: t.szilitorok@erasmusmc.nl

**Table 1. Cohen’s Kappa: disturbance noted**

|  |  | Electrophysiologist 2  Disturbance noted | |  |
| --- | --- | --- | --- | --- |
|  |  | No | Yes | Total |
| Electrophysiologist 1  Disturbance noted? | No | 167 | 3 | 170 |
|  | Yes | 0 | 4 | 4 |
|  | Total | 167 | 7 | 174 |

Cohen’s kappa was run to determine if there was agreement between the two electrophysiologists in electrogram disturbance evaluation. There was excellent agreement between the two observers (κ = 0.719, P = 0.000).

**Table 2. Cohen’s Kappa: disturbance severity score**

|  |  | Electrophysiologist 2  Disturbance severity score | | | |  |
| --- | --- | --- | --- | --- | --- | --- |
|  |  | None | Mild | Moderate | Severe | Total |
| Electrophysiologist 1  Disturbance severity score | None | 167 | 2 | 1 | 0 | 170 |
|  | Mild | 0 | 3 | 0 | 0 | 3 |
|  | Moderate | 0 | 0 | 0 | 1 | 1 |
|  | Total | 167 | 5 | 1 | 1 | 174 |

Cohen’s kappa was run to determine if there was agreement between the two electrophysiologists in electrogram disturbance severity scores. There was excellent agreement between the two observers (κ = 0.628, P = 0.000).

**Figure 1. ECM connection**

**
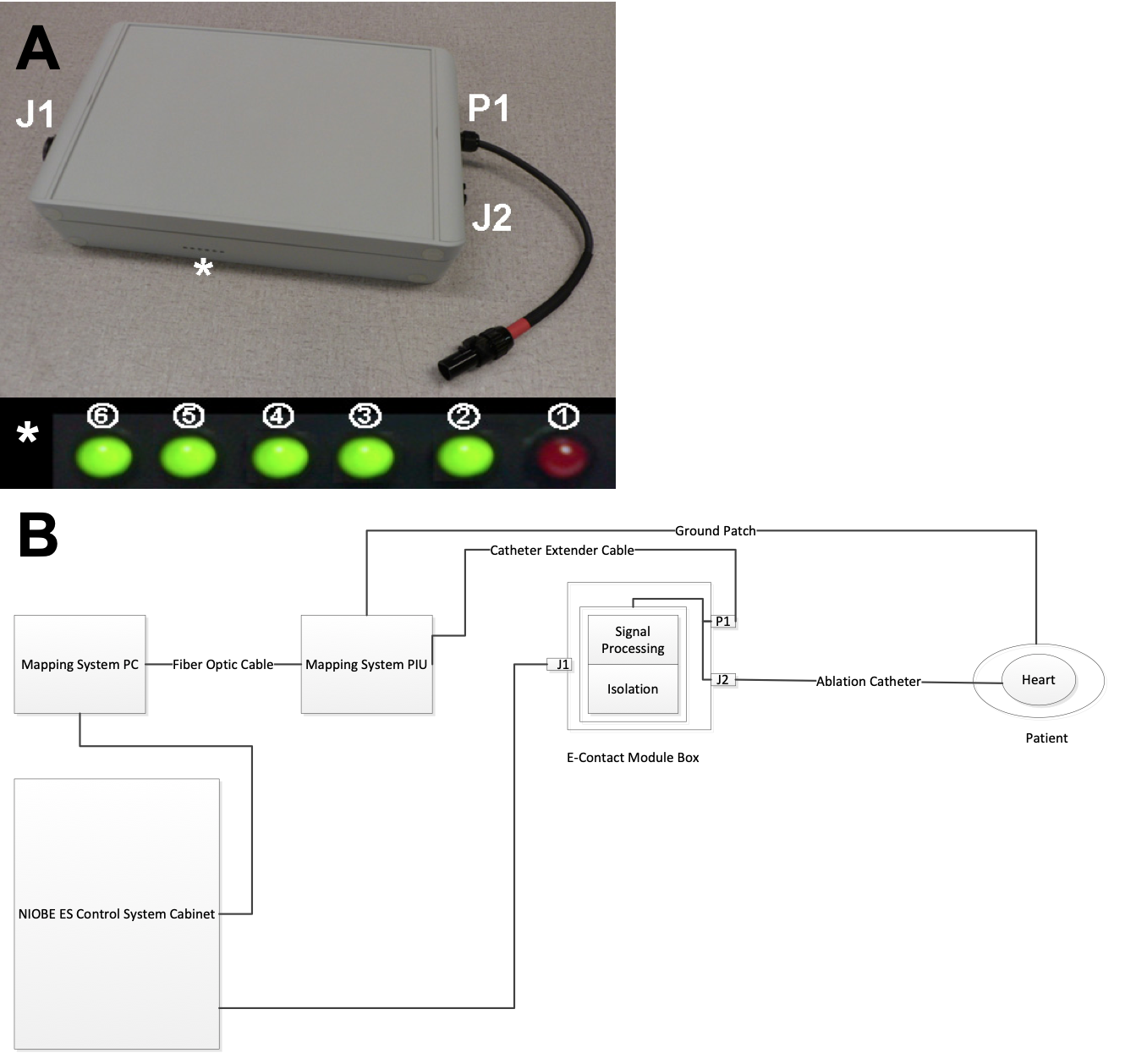
**

Figure 1A: The ECM box (hardware), which is to be connected to the Niobe ES System.

Figure 1B: Schematic presentation of how the ECM hardware box is connected to other systems. An Ethernet cable that provides power and communication for the ECM is connected from the port on the box (J1) to the Niobe ES System. Using the P1 port, the ECM box is connected to the mapping system PIU. The ablation catheter is connected to the ECM using the catheter port (J2). Once all connections are complete, all LED lights on the ECM box (marked with *) will illuminate green. This indicates that all signals are passed through unaltered while measuring the impedance on the electrodes of the ablation catheter.

*ECM = e-Contact Module*
